# Physical exercise speeds up motor timing

**DOI:** 10.3389/fpsyg.2013.00612

**Published:** 2013-09-11

**Authors:** Olga V. Sysoeva, Marc Wittmann, Andreas Mierau, Irina Polikanova, Heiko K. Strüder, Alexander Tonevitsky

**Affiliations:** ^1^The Center for Neurocognitive Research (MEG-Center), Moscow State University of Psychology and EducationMoscow, Russia; ^2^Laboratory of Human Higher Nervous Activity, Institute for Higher Nervous Activity and Neurophysiology, Russian Academy of SciencesMoscow, Russia; ^3^Department of Empirical and Analytical Psychophysics, Institute for Frontier Areas of Psychology and Mental HealthFreiburg, Germany; ^4^Institute of Movement and Neurosciences, German Sport University CologneCologne, Germany; ^5^Department of Physical Education, Lomonosov Moscow State UniversityMoscow, Russia

**Keywords:** physical exercise, motor timing, elite athletes, sport, cognition

## Abstract

This study aimed to examine effect of physical exercise on motor timing: personal, maximum and “once per second” tapping. The acute effect was examined by comparing the baseline tapping with that after acute exercise in 9 amateur athletes, 8 elite synchronous swimmers and 9 elite biathletes. Then the baseline tapping was compared among athletes of different sports and professional levels (15 elite biathletes, 27 elite cross-country skiers, 15 elite synchronous swimmers and 9 amateur wrestlers) with a control group (44 non-athletes) not involved in regular exercise to examine the sport-specific or long-term effects. Maximum and “once per second” tapping speed increased after acute physical exercise and were also faster in elite athletes compared to controls during the baseline condition. However, personal tapping tempo was not affected by exercise. In addition, physical exercise had no effects on the variability of the intertap interval. The accuracy of “once per second” tapping differentiates controls and amateur wrestlers from elite synchronous swimmers and skiers suggesting sport-specific adaptations to play a role. It is concluded that acute physical exercise selectively speeds up motor timing but does not affect its variability and accuracy, and this speeding-up is suggested to transfer into a long-term effect in elite athletes.

## Introduction

Physical exercise is known to have a wide range of effects on the body including the brain. For example, it has been shown that regional cerebral blood flow increases during dynamic exercise (Herholz et al., 1987). This is accompanied by alterations of opioidergic mechanisms (Boecker et al., 2008) as well as serotonergic (5-HT) (Strüder and Weicker, 2001a,b), noradrenergic (NA) and dopaminergic (DA) neurotransmitter systems activity (Meeusen and de Meirleir, 1995). Furthermore, dynamic exercise induces an increase in the plasma concentration of neural growth factors such as brain-derived neurotrophic factor as well as insulin-like growth factor 1, which are known to stimulate synaptogenesis and neurogenesis in the brain (Carro et al., 2000; Cotman and Berchtold, 2002; Cotman and Engesser-Cesar, 2002; Rojas Vega et al., 2006). These neurophysiological responses to exercise are accompanied by significant functional and behavioral changes comprising motor, perceptual, emotional and memory processes. For example, studies dealing with brain function during or after exercise reported shorter reaction times (Davranche and Audiffren, 2004; Davranche et al., 2006), improved sensorimotor adaptation (Mierau et al., 2009), reduced pain perception (Janal et al., 1984), improved mood states (to the point of euphoria) (Janal et al., 1984; Boecker et al., 2008; Schneider et al., 2009), reduced anxiety (Morgan, 1985; Petruzzello et al., 1991; Petruzzello and Landers, 1994), better memory performance (Erickson et al., 2011) as well as a facilitation of attentional and executive functions (Hillman et al., 2003, 2008; Weinstein et al., 2011). Other studies reported shorter estimates of duration (Vercruyssen et al., 1989; Schorr and Schroeder, 1991) and reduced lateness in coincidence-anticipation task (Fleury and Bard, 1987) as an effect of physical exercise. Recently it has been proposed that physiological changes of the body, as received and integrated in the insular cortex, are the basis for time perception (Craig, 2009; Wittmann, 2009). Thus, exercise-induced physiological changes of the body could have a strong impact on temporal processes including motor timing and this topic has not been studied previously.

Neuropsychological studies with pharmacological agents and examining patients with brain lesions suggest that distinct brain networks are associated with the voluntarily-chosen (or personal) speed and with movements at maximum pace (Wittmann et al., 2001, 2007). That is, movements with a frequency of 1–2 Hz are under voluntary control and allow the collection of somatosensory information (feedback control), while movements at maximum speed with frequencies of 5 Hz are under coarse pre-attentive control (feed-forward control) (Kunesch et al., 1989; Peters, 1989; Wittmann et al., 1999). Several studies show how these two functional modes are independently affected. Whereas maximum tapping speed was unaffected by administration of the hallucinogenic agent psilocybin, personal tapping tempo was significantly slowed down during peak effects of psilocybin (Wittmann et al., 2007). Moreover, personal tapping was slowed down in patients with left-hemispheric cortical lesions; maximum tapping speed was not influenced by cortical lesions to either side of the brain (Wittmann et al., 2001). That is, in contrast to the maximum tapping tempo personal tapping can be seen as reflecting the voluntarily-chosen internal tempo of an individual, potentially associated with an internal pacemaker (Vanneste et al., 2001). Subjective time pacing can be assessed by tapping at a pace of “once per second,” which taps into the ability to produce the conventional time unit by activating the internal representation of one second. Previous studies point to the relationship between the production of short-time intervals (up to 1–2 s) and dopaminergic activity (Portnova et al., 2007; Coull et al., 2011).

The aim of the current study was to examine the effect of physical exercise on different aspects of internally generated motor timing parameters such as personal tapping, maximum tapping and “once per second” tapping. Therefore, the three tapping tasks were performed at baseline condition (BL) and after an acute bout of physical exercise (AE). Recent meta-analyses showed that the effect of acute exercise on cognitive performance is significantly modulated by numerous factors including exercise intensity, type and duration of the exercise bout, time elapsed after the exercise, as well as the subject's fitness/professional level (Tomporowski, 2003; Lambourne and Tomporowski, 2010; Chang et al., 2012). Our study included some of these moderators to examine if this will change the potential effects of acute exercise on motor timing. We studied three groups of athletes: elite cross-country skiers, synchronous swimmers, and semi-professional wrestlers, performing three different types of exercises: a graded running test (GRT) on a treadmill until subjective exhaustion (biathletes), and two types of intense practice (synchronous swimming and combat wrestling). It was intended to examine whether acute exercise would produce differential effects on the three motor timing tasks and whether the level of involvement in sports (professional level) or type of exercise would modulate this effect. We hypothesized that physical exercise will generally speed up motor timing, but the effects on different tapping tasks might not be interrelated due to physiological and neuroanatomical differences underlying these three tapping tasks.

Our second aim was to examine long-term or sport-specific effects of physical exercise on motor timing. It has been shown that expert athletes outperformed non-experts in many, especially domain specific, cognitive and perceptual tasks (Mann et al., 2007). In our study, the baseline tapping conditions were compared among athletes of different sports and professional levels with a control group not involved in regular exercise. We hypothesized that continuous acute exercise effects on motor timing transferred to long-term effects in elite athletes. In addition, within-athletes differences depending on the type of sport were expected. In particular, it was hypothesized that synchronous swimmers will have more accurate “once per second” intervals because they are trained to produce subtly timed movements.

## Materials and methods

### Participants

Fifty-seven elite athletes from Russian Olympic teams (15 biathletes: 5 females/10 males, age 26 ± 2 years; 27 cross-country skiers: 9 females/18 males, age 25 ± 5 years; 15 synchronous swimmers: all females, age 21 ± 3 years), 9 amateur/semiprofessional wrestlers from the university freestyle wrestling group (all males, age 19 ± 1 years) and 44 control non-athlete participants, who were not involved in regular sport activities (all males, age 24 ± 6 years) participated in the study. There was no significant age difference between the groups (*p* > 0.3). All participants gave their informed consent after the nature of the study was explained to them. The study was approved by the Institutional Review Board.

### Experimental tasks

Participants performed three types of tapping tests with their index finger of the right hand in the following order: personal tapping speed, maximum tapping and tapping “once per second.” Thus, we focused on “internal” modes of tapping behavior and did not study timing accuracy to external stimulation such as regular beats. In the personal (or self-paced) tapping task participants had to press a button repeatedly at their preferred rate; in the maximum tapping task participants pressed a button at maximum rate; in the “once per second” tapping condition participants were asked to press a button at a rate of once per second. No cues were provided to the participants about the objective second interval. There was no watch or clock visible on the computer screen or in the room. The participants were encouraged to rely on their inner sense of temporal intervals. No feedback about their performance was provided. These precautions were undertaken to activate an internal representation of a 1 s duration. The task was performed on a notebook (ASUS X51 RL) or stationary computer (Dell). E-prime (version 1.2) was used as presentation software. For the synchronous swimmers the NS-PsychoTest (NeuroSoft) was used and instead of a computer button press participants used a special electronic device (rubber pad with electronic pen) connected to the computer via USB. For elite athletes each tapping task was terminated after 30 s. For wrestlers and the control group the personal tapping task stopped when 90 responses were collected, “once per second” tapping stopped when 50 responses were collected and the maximum tapping task stopped after 60 s. All participants were right handed and used their right hand to perform the task.

### Experimental procedure

All participants performed the three experimental tasks at baseline condition (BL). With the exception of the cross-country skiers all athletes groups were also asked to perform the three tasks after exercise (AE). Due to technical/organizational factors we were able to get AE data only from a number of participants: 9 biathletes, 8 synchronous swimmers and 9 wrestlers.

For the wrestlers and the synchronous swimmers the exercise was their regular training sessions: a 30 min combat wrestling exercise with a mean heart rate of 160 ± 7 beats/min during exercise, and 2 h swimming practice, with AE mean heart rate of 70 ± 11 beats/min, respectively. Biathletes performed a GRT on a treadmill in our laboratory with an initial running velocity of 12.6 km/h (men) and 9.0 km/h (women), increasing by 1.8 km/h every 3 min until subjective exhaustion (AE mean heart rate: 91 ± 9 beats/min; average duration of test: 16 min). For the wrestlers and the synchronous swimmers, whose training location was based in Moscow, BL and AE assessments were conducted on two separate days in a counterbalanced order across participants. However, this was not possible for the biathletes since they visited our laboratory only on one specific day for a routine medical and physical examination. Therefore, both the BL and AE conditions were assessed within one day; 6 min before and 3–10 min after GRT. The biathletes and the synchronous swimmers performed AE condition within 20 min after exercises. Cross-country skiers and non-athletes controls performed the task only once before any physical exercise. The procedure took place in our laboratory.

### Statistical analysis

Mean intertap intervals and intertrial variability (SD) were calculated for each subject. The intertrial variability was correlated with the mean tapping interval (*r* = 0.57, 0.39 and 0.76 for “once per second,” maximum and personal tapping, respectively) and these correlations confirmed the known increase in variability with increasing tapping intervals. To account for this relationship, an adjusted variability measure (intertrial variability divided by the mean intertap interval) was calculated and studied for each experimental group and task. In addition, we determined a measure of accuracy for the “once per second” tapping as the absolute difference between mean subjects' intertap interval and the objective second.

To study the effects of acute physical exercise on different athlete groups a mixed repeated measures ANOVA with Condition (BL and AE) as a within-subject variable and Group (elite biathletes, elite synchronous swimmers and amateur wrestlers) as the between-participants variable was performed separately for the intertap interval of each tapping task. The adjusted variability measure is comparable across tapping types and therefore, it was analyzed within one repeated measures ANOVA with Condition (BL and AE) and Tapping Type (“once per second,” maximum and personal) as a within-subject variables and Group (elite biathletes, elite synchronous swimmers and amateur wrestlers) as the between-participants variable. The relationship between the acute effect of physical exercise (difference scores: AE - BL) on different tapping tasks was examined by Pearson correlations.

To study the effect of different sport types and professional levels a univariate ANOVA with Group (elite biathletes, elite skiers, elite synchronous swimmers, amateur wrestlers and non-athlete controls) as fixed factor was applied to each of the three tapping tasks' baseline condition. Significant main effects and interactions were further analyzed using the Fischer's Least Significant Difference (LSD) *post-hoc* test.

The reliability of tests scores was examined by Cronbach's alpha method, comparing scores obtained in BL and AE conditions, and was 0.99, 0.99 and 0.79 for “once per second,” maximal and personal intertap interval, respectively.

## Results

### Acute effect of physical exercise

The results for the acute effect of physical exercise on motor timing are summarized in Table 1. The mixed ANOVA revealed a significant main effect for Condition and Group for the tapping “once per second” [Condition: *F*_(1, 23)_ = 18.23, *p* < 0.001, η^2^ = 0.442; Group: *F*_(2, 23)_ = 13.12, *p* < 0.0001, η^2^ = 0.533] and the maximum tapping [Condition: *F*_(2, 23)_ = 17.67, *p* < 0.001, η^2^ = 0.434; Group: *F*_(2, 23)_ =5.2, *p* < 0.05, η^2^ = 0.311] with no significant Group × Condition interaction [*F*_(2, 23)_ = 1.54, *p* = 0.23, η^2^ = 0.118 and *F*_(2, 23)_ = 0.12, *p* = 0.89, η^2^ = 0.010], respectively. The average “once per second” and maximum tapping interval decreased after physical exercise for all groups, reflecting an increase in tapping speed. *Post-hoc* analysis following Group effect revealed that both maximum (*p* < 0.01) and “once per second” tapping (*p* < 0.01) was significantly faster in biathletes compared to wrestlers and the “once per second” tapping intervals were shorter in biathletes compared to synchronous swimmers (*p* < 0.01). There were no significant effects for personal tapping [Condition: *F*_(1, 23)_ = 0.41, *p* = 0.53, η^2^ = 0.017; Group: *F*_(2, 23)_ = 0.94, *p* = 0.40, η^2^ = 0.076; Group × Condition interaction: *F*_(2, 23)_ = 1.54, *p* = 0.23, η^2^ = 0.118]. The different effects of exercise on the tapping tasks did not correlate with each other (*p* > 0.3).

**Table 1:** **Tapping interval and its within subject variability during baseline (BL) and after exercise (AE) for three groups of athletes (M ± SD)**.

	***n***	**“Once per second”**						**Maximum tapping**				**Personal tapping**			
		**Interval (ms)**		**Variability (%)**		**Accuracy (ms)**		**Interval (ms)**		**Variability (%)**		**Interval (ms)**		**Variability (%)**	
		**BL**	**AE**	**BL**	**AE**	**BL**	**AE**	**BL**	**AE**	**BL**	**AE**	**BL**	**AE**	**BL**	**AE**
Biathletes	9	837 ± 125	694 ± 135	6.1 ± 2.5	6.5 ± 2.1	163 ± 125	305 ± 135	153 ± 10	142 ± 10	13.2 ± 6.5	11.5 ± 5.3	620 ± 299	579 ± 137	7.8 ± 4.3	6.7 ± 2.6
Synchronous swimmers	8	1115 ± 185	957 ± 191	8.9 ± 5.9	8.0 ± 5.0	154 ± 103	148 ± 114	164 ± 18	154 ± 19	17.3 ± 6.8	21.7 ± 14.7	857 ± 746	1227 ± 1552	19.7 ± 18.0	12.6 ± 5.7
Amateur wrestlers	9	1219 ± 252	1028 ± 61	7.8 ± 2.7	6.8 ± 2.8	264 ± 197	57 ± 31	176 ± 22	167 ± 20	21.8 ± 23.9	17.9 ± 15.0	952 ± 608	850 ± 350	15.4 ± 13.6	15.0 ± 12.4

For the adjusted variability there were neither significant effects of Condition [*F*_(1, 23)_ = 1.17, *p* = 0.29, η^2^ = 0.048], nor of Group [*F*_(1, 23)_ = 2.98, *p* = 0.07, η^2^ = 0.206] but a significant effect of Tapping Types [*F*_(2, 23)_ = 7.78, *p* = 0.002, η^2^ = 0.253, Greenhouse-Geisser correction applied]. Student *t*-test analysis followed the significant effect of Tapping Types revealed significantly lower variability in “once per second” tapping (7.3) compared to personal [12.8: *t*_(25)_ = 2.75, *p* = 0.11] and maximum tapping [17.2: *t*_(25)_ = 3.72, *p* = 0.001]. There was no difference in variability between maximum and personal tapping [*t*_(25)_ = 1.64, *p* = 0.15]. There were also no significant interactions indicating that physical exercise did not affect the adjusted variability in neither of the tapping task in any sport groups [*p* > 0.17, η^2^ < 0.128].

For the accuracy in “once per second” tapping, there was neither an effect of Group [*F*_(1, 23)_ = 1.67, *p* = 0.21, η^2^ = 0.126] nor Condition [*F*_(1, 23)_ = 0.67, *p* = 0.42, η^2^ = 0.028] but a significant Group × Condition interaction [*F*_(1, 23)_ = 12.62, *p* < 0.001, η^2^ = 0.523]. Following this significant interaction effect, *t*-test analysis revealed that accuracy significantly decreased in biathletes [*t*_(8)_ = 2.92, *p* = 0.02], increased in amateur wrestlers [*t*_(8)_ = 3.35, *p* = 0.01], and did not change in synchronous swimmers [*t*_(7)_ = 0.19, *p* = 0.85] following exercise.

### Long-term effect of physical exercise

The results for the long-term effect of physical exercise on motor timing presented in Table 2. An univariate ANOVA showed a significant Group effect for both, the “once per second” [*F*_(4, 110)_ = 3.76, *p* < 0.01, η^2^ = 0.125] and the maximum tapping [*F*_(4, 110)_ = 7.22, *p* < 0.0001, η^2^ = 0.216], but not for personal tapping [*F*_(4, 110)_ = 1.91, *p* = 0.11, η^2^ = 0.068]. For “once per second,” tapping the LSD *post-hoc* test revealed that each of the elite athlete groups (skiers, biathletes and synchronous swimmers) had significantly shorter tapping intervals (indicating a higher speed) than amateur wrestlers and the non-athlete control group (*p* < 0.05). However, there was no significant difference between the three elite athlete groups, and the amateur wrestlers did not significantly differ from the non-athlete group (*p* > 0.05). For maximum tapping, the *post-hoc* analyses revealed that all elite athlete groups (skiers, synchronous swimmers and biathletes) had significantly shorter tapping intervals than the non-athlete group (*p* < 0.05). The amateur wrestlers did not differ significantly either from the non-athlete or from the elite athlete groups (*p* > 0.1). Again, there were no significant effects for personal tapping (*p* > 0.1). There was a significant effect of Group on the accuracy of “once per second” interval [*F*_(4, 110)_ = 2.96, *p* = 0.23, η^2^ = 0.101]. As revealed by the *post-hoc* test, non-athletes and amateur wrestlers had a significantly less accurate representation of one second than elite synchronous swimmers and skiers (*p* < 0.05).

**Table 2:** **Tapping interval (ms) in the baseline condition for different groups of athletes and non-athlete controls**.

	***n***	**Once per second**	**Accuracy**	**Maximum tapping**	**Personal tapping**
		**M** ± **SD**	**M** ± **SD**	**M** ± **SD**	**M** ± **SD**
Biathletes	15	922 ± 198	175 ± 114	157 ± 11	635 ± 280
Skiers	27	971 ± 171	137 ± 104	150 ± 17	788 ± 450
Synchronous swimmers	15	1026 ± 153	122 ± 98	166 ± 15	1037 ± 694
Amateur wrestlers	9	1219 ± 252	264 ± 197	176 ± 22	952 ± 608
Non-athletes	44	1087 ± 294	235 ± 194	198 ± 60	1172 ± 759

No gender differences were found for “once per second” and personal tapping, when compared separately within cross-country skiers and biathletes (*p* > 0.3). Maximal tapping speed was higher in males (145 ± 13 ms) than females (161 ± 18 ms) in the cross-country skiers group only [*t*_(25)_ = 2.67, *p* = 0.01; biathletes: *p* > 0.3]. These results provide evidence that the group differences as found in our study cannot be accounted for by between-group gender heterogeneity.

## Discussion

The present study shows that motor timing is affected by physical exercise. Specifically, a significant increase in speed in maximum “once per second” tapping was detected after acute physical exercise. The effect was present for all experimental groups, performing different types of exercise. This fact provides support for a robust effect of acute exercise on motor timing, independent of the subject's professional level and exercise variables such as type, intensity and duration. To the best of our knowledge this is the first study that shows significant effects of acute physical exercise on basic parameters such as the temporal constraints in motor performance. However, it is worth noting that physical exercise did not affect the variability of the intertap interval.

Consistent with our hypothesis about the transfer of continuous acute exercise effects to long-term effects, we found that the group of elite athletes (biathletes, skiers, synchronous swimmers) have an increased maximum and “once per second” tapping speed as compared to the control group that was not involved in regular sports activities. The elite athletes groups did not differ from one another in tapping scores, suggesting that the long-term effect of physical exercise on motor timing is not domain-specific. The amateur wrestlers group was similar to the control group in “once per second” tapping but did not differ from the other elite athletes in the maximum tapping speed. The results for the amateur wrestler group might not only be explained by differences in the professional level of sport but also by the type of sport (wrestling), which has some important differences as compared to biathlon, skiing and synchronous swimming. Some procedural differences such as the duration of the tapping tasks might also influence the results. Unfortunately, our study could not fully disentangle these possibilities. Nonetheless, taken together with the general pattern of results, where all elite athletes show a similarly increased maximum tapping speed and are faster in the “once per second” tapping, the professional level explanation seems more probable. Therefore, the data suggest that physical exercises have long-term effect on motor timing and change the representation of the conventional time unit of one second. However, this explanation should be considered with caution since the motor timing parameters in elite athletes might also be related to other factors, such as initial genetic differences between the groups (Sysoeva et al., 2009; Tucker and Collins, 2012).

Importantly, the effect of physical exercise on the three types of tapping was different. Personal tapping was not modulated by physical exercise and acute effects of exercise on maximum and “once per second” tapping did not correlate with each other. This fact provides evidence for differential processes involved in these tapping tasks. Two pre-attentive processes involved in motor timing are modulated by physical exercise: general motor speed, as assessed in the maximum tapping condition, and a sense of short duration, assessed by the “once per second” tapping condition. More evidence for the claim that differential mechanisms are involved in maximum and “once per second” tapping can be derived from the performance of the amateur athlete group. It is different as compared with the elite athletes in “once per second” tapping, but not in maximum tapping speed. Additionally, only “once per second” but not maximum tapping intervals were significantly shorter in biathletes compared to synchronous swimmers following acute exercise. The acute within-participant effects on maximum tapping can probably be attributed to an increased arousal level after exercise leading to an increase in tapping frequency due to a facilitation of motor processes (Davranche et al., 2006). In the personal tapping mode more self-control in motor timing might have prevented a speed-up. This result adds to the increasing number of studies showing that different modes of motor timing are governed by different brain systems (Wittmann et al., 2001, 2007). Specifically, personal tapping tempo is under continuous voluntary control with a self-paced speed, whereas maximum tapping speed is driven by automatic feed-forward control processes. In addition, the “once per second” tapping speed relates to an inner representation of a conventional and highly trained time unit. Noteworthy, the performance in “once per second” tapping was less variable than in other tapping types, indicating the relative stability of the representation of conventional time unit. Our study suggests that physical exercise influences this internal representation of time. One potential mechanism of such an influence includes brain processes related to body awareness and the dopaminergic modulatory system, which is affected by physical exercise (Meeusen and de Meirleir, 1995) and involved in duration representation (Portnova et al., 2007; Coull et al., 2011).

The long-term effects of physical exercise in maximum tapping task can be potentially explained as an increased efficiency in motor execution in elite athletes as compared to controls. More interesting and novel results come from “once per second” tapping, where previously mentioned interpretations are not sufficient. Both acute and long-term effects of facilitation in “once per second” tapping might be related to a central timing process, and can be explained as an increase in internal timing pacemaker speed (Burle and Casini, 2001), or as relative overrepresentation of duration due to an increase in the awareness of body states (Craig, 2009; Wittmann, 2009), which lead to a lengthening in subjective time. This interpretation is consistent with previous reports of shorter duration productions (which are equivalent to duration overestimations) in the range of seconds as an effect of physical exercise (Vercruyssen et al., 1989; Schorr and Schroeder, 1991). According to a recent study (Jones et al., 2011), the speeding up of subjective time by click trains affects information-processing rate in choice reaction, mental arithmetic and memory tasks. Therefore, speeding up of subjective time by physical exercise, found in our study, may at least partially, explain the previously reported effects of physical exercise on cognitive functions (reviewed in Tomporowski, 2003; Lambourne and Tomporowski, 2010; Chang et al., 2012).

In line with our hypothesis, synchronous swimmer had the most accurate internal representation of one second, which is suggested to be due to their intensive training to synchronize their movements with objective events in music. Therefore, the objectivity of their representation is crucial for their performance. Interestingly, cross-country skiers were not significantly different in accuracy than synchronous swimmers but significantly better than amateur wrestlers and non-athletes, suggesting that accuracy in “once per second” tapping might be related to more general physical training effects rather than to synchronous swimming *per se*. Elite biathletes did not differ from non-athletes showing the worst accuracy among the elite athletes group. This effect might be explained by the fact that biathletes are more sensitive to their inner body changes as a result of specific demands during competition such as shooting in relation to their bodily processes such as breathing rate and heart beats (Helin et al., 1987; Konttinen et al., 2003). Therefore, they are trained to adjust their motor behavior to inner processes whereas objective duration representation is not as crucial for their performance as it is for synchronous swimmers. Unfortunately, there is a substantial gap in research concerning the relationship between physical exercise and the internal representation of time. Therefore, the aforementioned aspects should be considered as hypothesis to be tested in the future rather than conclusive interpretations. However, our general assumption that the athletes' brain is characterized by both, sport-specific and rather generalized (physical activity-related) functional adaptations is supported by numerous studies (Babiloni et al., 2010; see Nakata et al., 2010 for an overview).

Many interpretations of our study results have to be regarded with caution. For example, long-term effects of exercise on motor timing can only be rigorously assessed with a longitudinal study design. However, regarding short-term effects of exercise we present for the first time a study that assesses internally generated tapping performance in athletes. The findings of specific effects on maximum and “once per second” but not on personal tapping speed need additional empirical support and variations in study design.

## Conclusions

The main finding of the present study is that acute physical exercise speeds up maximum and “once per second” tapping and that this effect may potentially transfer into a long-term effect in elite athletes. The effects on the two tapping tasks are related to two independent processing components: general motor speed and interval timing. This decomposition of motor timing performance into a more peripheral motor and a more central timing related components has been introduced in the model by Wing and Kristofferson (1973), which is usually applied in the analysis of synchronized tapping to external stimuli. In our tapping tasks of internally generated tapping speed these two components are allocated to two different tasks. One strong interpretation that one would have to follow up in future studies would be that physical exercise influences this internal representation of time.

### Conflict of interest statement

The authors declare that the research was conducted in the absence of any commercial or financial relationships that could be construed as a potential conflict of interest.
